# Cerebral Arterial Stenoses and Stroke: Novel Features of Aicardi-Goutières Syndrome Caused by the Arg164X Mutation in *SAMHD1* Are Associated with Altered Cytokine Expression

**DOI:** 10.1002/humu.21357

**Published:** 2010-11

**Authors:** Holger Thiele, Marcel du Moulin, Katarzyna Barczyk, Christel George, Wolfram Schwindt, Gudrun Nürnberg, Michael Frosch, Gerhard Kurlemann, Johannes Roth, Peter Nürnberg, Frank Rutsch

**Affiliations:** 1 Cologne Center for Genomics, University of Cologne50931 Cologne, Germany; 2 Department of General Pediatrics, Münster University Children's Hospital48149 Münster, Germany; 3 Institute for Immunology, Münster University Hospital48149 Münster, Germany; 4 Department of Clinical Radiology, Münster University Hospital48149 Müünster, Germany

**Keywords:** cytokine, cerebrovascular stenosis, stroke, *SAMHD1*, interleukin-8, immune response

## Abstract

Aicardi-Goutières syndrome (AGS) is a rare inborn multisystemic disease, resembling intrauterine viral infection and resulting in psychomotor retardation, spasticity and chilblain-like skin lesions. Diagnostic criteria include intracerebral calcifications and elevated interferon-alpha and pterin levels in cerebrospinal fluid (CSF). We report on four adult siblings with unknown neurodegenerative disease presenting with cerebrovascular stenoses, stroke and glaucoma in childhood, two of whom died at the age of 40 and 29 years. Genome-wide homozygosity mapping identified 170 candidate genes embedded in a common haplotype of 8Mb on chromosome 20q11-13. Next generation sequencing of the entire region identified the c.490C>T (p.Arg164X) mutation in *SAMHD1*, a gene most recently described in AGS, on both alleles in all affected siblings. Clinical diagnosis of AGS was then confirmed by demonstrating intracerebral calcifications on cranial computed tomography in all siblings and elevated pterin levels in CSF in three of them. In patient fibroblasts, lack of SAMHD1 protein expression was associated with increased basal expression of *IL8*, while stimulated expression of *IFNB1* was reduced. We conclude that cerebrovascular stenoses and stroke associated with the Arg164X mutation in *SAMHD1* extend the phenotypic spectrum of AGS. The observed vascular changes most likely reflect a vasculitis caused by dysregulated inflammatory stress response. © 2010 Wiley-Liss, Inc.

## INTRODUCTION

Aicardi-Goutières syndrome (AGS; MIM# 225750) was first described in 1984 as an early onset familial encephalopathy inherited autosomal recessively (Aicardi and Goutières, 1984). It is a multisystemic disorder resembling intrauterine viral infection (Goutières, 2005).

Patients present with psychomotor retardation, progressive microcephaly, dystonic posturing and spasticity as well as chilblain-like skin lesions (Tolmie et al., 1995). Congenital glaucoma may be present (Crow et al., 2000). The syndrome manifests in infancy and death often occurs early in childhood in a state of decerebration. However, milder forms and survival into early adulthood have been reported (Goutières et al., 1998; Rice et al. 2009).

Intracerebral calcifications, specifically at the site of the basal ganglia, cerebral atrophy, and leukodystrophy detected by cranial computed tomography (CT) scan are the most important characteristics (Goutières, 2005). Other diagnostic criteria include chronic lymphocytosis in cerebrospinal fluid (CSF) (Goutières, 2005) and elevated levels of interferon-α (IFN-α) in CSF and serum (Lebon et al., 1988). However, elevation of IFN-α levels diminishes with time (Goutières et al., 1998) or may be absent (Blau et al., 2003; Wassmer et al. 2009). In such cases, elevated pterins in CSF can be a useful diagnostic marker (Blau et al., 2003; Wassmer et al. 2009). Furthermore, thrombocytopenia and elevated transaminases may be found (Goutières et al., 1998).

Mutations in several genes have been found to be associated with AGS including *TREX1*, a gene on chromosome 3p31.21 encoding a DNA exonuclease (Crow et al., 2006a). Mutations in three genes encoding the ribonuclease H2 enzyme complex, *RNASEH2A* on 19p13.13, *RNASEH2B* on 13q14.3, and *RNASEH2C* on 11q13.2, have also been described in the pathogenesis of AGS (Crow et al., 2006b). Early onset of symptoms is associated with *TREX1* mutations, while mutations in *RNASEH2B* lead to a late-onset presentation, a milder phenotype and reduced mortality (Rice et al., 2007). Most recently, mutations in *SAMHD1* on 20q11 were identified in AGS (Rice et al., 2009) and phenotypic overlap with systemic lupus erythematosus has been described (Ramantani et al., 2010).

We report on four affected siblings, two males and two females, born to consanguineous parents of Turkish origin (Table 1). The patients presented with an unknown multisystemic, neurodegenerative disorder. Their main clinical symptoms, including stenoses of intracranial vessels, stroke and glaucoma, could not be linked to any previously described syndrome. Therefore, using genomic DNA from the four affected siblings, we performed genome-wide homozygosity mapping and next generation sequencing and identified a mutation in *SAMHD1* in all four patients. As this gene has been most recently described in AGS (Rice et al., 2009), we retrospectively confirmed the diagnosis by demonstrating intracerebral calcifications on CT scans in all four patients and elevated pterins in CSF in three of them.

**Table 1:** Clinical Features of Patients with the Arg164X Mutation in *SAMHD1*

	Patient 1	Patient 2	Patient 3	Patient 4
**Brain**				
Intracerebral calcifications	+	+	+	+
Cerebral atrophy	+	+	-	+
Stenosis of intracerebral vessels	+	+	+	+
Stroke	-	-	-	+
**Neurology**				
Spasticity	+	+	-	+
Dystonic posturing	+	+	-	+
Seizures	-	+	-	-
**Development**				
Psychomotor retardation	+	+	(+)	+
**Eyes**				
Glaucoma	+	+	+	+
Cataract	+	+	-	-
**Skin**				
Chilblain-like lesions	-	+	+	+
**Endocrinology**				
Hypothyroidism	-	-	+	+
Precocious puberty	+	+	+	+
**Musculoskeletal**				
Joint contractures	?	+	+	+
**Growth**				
Short stature	+	+	+	+
Microcephaly	?	+	-	+
**Survival**				
Age at death	40 years	29 years	Alive at 25	Alive at 20
**Laboratory Findings**				
Elevated transaminases	?	+	(+)	(+)
Thrombocytopenia	?	-	-	-
Lymphocytosis in CSF	+	(+)	-	-
Interferon-α in CSF	/	/	-	-
Interferon-α in serum	/	/	-	-
Elevated pterins in CSF	/	+	+	+

Data are indicated as follows: +, present; (+), mildly present; -, absent; /, not analysed; ?, data not available. Abbreviations: CSF, cerebrospinal fluid

## CASE REPORTS

Patients were included in the study after obtaining written informed consent and the study was approved by the ethical committee of Münster University. Clinical findings of all patients are summarized in Table 1.

### Patient 1

The male patient was the second child born to consanguineous parents originating from Turkey. Six siblings (three male and three female) are asymptomatic. At one year of age he was diagnosed with meningitis.

He was of small stature and had reduced mental capabilities. His communication skills were sufficient, and cooperation was adequate. He was tetraspastic and mobile with support of a walking aide only. He developed glaucoma and cataract on his left eye, eventually leading to blindness. Furthermore, precocious puberty had been noted. His skin was unremarkable. Thyroid hormone levels were normal.

Magnetic resonance (MR) angiography, performed at age 37, showed irregular vascular structures of the left medial cerebral artery.

At age 40, a CT scan showed symmetrical calcifications of the basal ganglia and of the white matter as well as global cerebral atrophy. CSF analysis revealed an increased cell count and elevated protein and lactate levels. Because of pathological MR findings in combination with the CSF study, the patient was thought to suffer from tuberculous meningitis and was treated with tuberculostatic drugs for 60 days. The clinical course was complicated by respiratory insufficiency, secondary to nosocomial pneumonia. The patient later developed acute respiratory distress syndrome and acute renal failure. He died of severe sepsis at 40 years of age.

### Patient 2

The male patient was the fifth child of the family. He was born after an uneventful pregnancy (birth weight was 3,300 g, length 50 cm, head circumference 35 cm, Apgar 10/10/10). Congenital glaucoma was diagnosed.

At 3 months of age, motor development was delayed. There was no fixation at the age of 1 year and buphthalmos was noticed. At 2 ½ years of age, he could move using all four extremities and was able to sit without support. Walking was possible with an aide only. The patient suffered from a seizure with transient postconvulsive hemiparesis at 3 years of age. Another seizure occurred at 5 years of age. He lost speech at the age of 6 years and communicated using signs.

Symmetrical calcifications of the basal ganglia as well as cerebral atrophy were evident on cerebral CT at the age of 8 years. A maculous, erythematous, bluish-coloured skin lesion was first noticed on the left foot. Also, precocious puberty was noted.

At the age of 20 years, the patient was severely dystrophic with a height of 122 cm (< 3^rd^ centile) and a weight of 20 kg (< 3^rd^ centile). Additionally, multiple small cervical fibromas of the skin were present.

At age 22, mild lymphocytosis and increased protein levels were found in CSF. IFN-α was not analysed; CSF pterin levels were elevated.

Cataract of the left side was diagnosed at the of age 25 years.

A cranial magnetic resonance imaging (MRI) scan, performed at the age of 25 years, revealed marked stenoses of the supraaortic vessels. The internal carotid arteries on both sides and the arteries of the Circulus Willisii could only hardly be detected.

Because of a butterfly rash, chronic arthropathy and contractures, an undefined encephalopathy and positive serum antibodies against double-stranded DNA, systemic lupus erythematosus was suspected at this time and the patient was treated with immunosuppressants for several years. Under this regimen, he developed pneumonia and intracerebral hemorrhage ultimately leading to death at the age of 29 years. Shortly before the incident, he had recurrent skin ulcers and was treated with prostaglandin infusions. It can be suspected that this therapy contributed to the bleeding.

### Patient 3

This female patient is the eighth child of the family. She was born at 38 weeks of gestation after an uneventful pregnancy (weight 3,600 g, length 51 cm, head circumference 35 cm, Apgar 9/10/10).

Symptoms were first noticed at age 4, when she presented with polyarthritis and inflammation of the eyes.

With 9 years of age, she complained of increasing, non-tender restriction of movement of the fingers, elbows, hips, knees, and feet. Later, pain and contractures developed. She had reduced vision and glaucoma of both eyes. At that time, precocious puberty was noted.

At age 12, she developed erythema of both forearms after exposure to sun. Additionally, she was diagnosed with primary hypothyroidism.

At age 16, her feet were deformed, showed signs of arthrosis and were increasingly painful. She also suffered from gonarthrosis.

Similar to her siblings, she has a short stature. At age 17, her height was 136 cm (< 3^rd^ percentile) and her weight was 48 kg (3^rd^ percentile).

**Figure 1:**
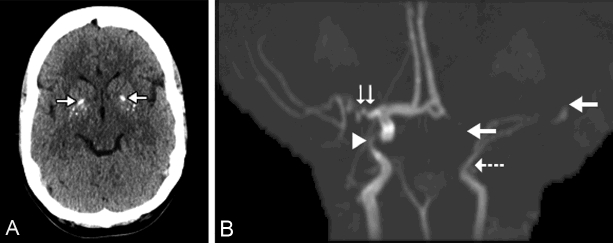
Radio logic findings in two patients homozygous for the Arg164X mutation in *SAMHD1*. (A) Patient 3 at age 25 years: Calcifications (arrows) in the basal ganglia (CT). (B) Patient 4 at age 14 years: MR angiography showing stenosis of the left distal internal carotid artery (arrow with dashed line) and discontinuation of the left medial cerebral artery (upper full arrow). The A1 segment of the left anterior cerebral artery is not detectable (lower full arrow). On the right, severe stenoses of the internal carotid artery (white arrowhead) and of the proximal part of the medial cerebral artery (double vertical arrow).

A CT scan, performed at age 25, showed bilateral calcifications of the basal ganglia (Figure 1A). At that time, CSF showed no pleocytosis. IFN-α was not elevated in CSF or serum. Pterin levels in CSF were increased (neopterin 93.3 nmol/l [9-20], biopterin 61.4 nmol/l [10-30]). MR angiography showed a fetal origin of the left posterior cerebral artery with a narrow segment without any pathological relevance. No inflammatory changes were seen on MRI of her right hand. Liver enzymes were mildly elevated (GOT 37 U/l [10-35], GPT 46 U/l [10-35], γ-GT61U/l[<39]).

Mental capabilities are mildly impaired. The patient is able to communicate well and properly. She currently works as a waitress.

### Patient 4

The female patient is the ninth child of the family. She was delivered by cesarian section because of intrauterine tachyarrhythmia at 36 weeks of gestation (birth weight 3,200 g, length 53 cm, head circumference 34 cm, Apgar 6/9/9).

Since early infancy, she suffered from spasticity of the arms and legs. An ophthalmological investigation in the first days of life revealed no signs of congenital glaucoma. A cranial CT scan at the age of 2 years showed intracerebral calcifications and oligoclonal bands were found in CSF. She started crawling between age 3 and 4 years and was able to sit without support between age 4 and 5. She never developed expressive language, but she was able to understand language. Since age 8, she has been wheelchair dependent.

During the first six years of life, she had recurrent episodes of dermatitis of the flexor sides of the extremities. On the palm of her hands, she developed plaque-like lesions. Her right toe showed necrosis. Calcinosis cutis with axillary nodules was also noticed.

At age 8, she was diagnosed with precocious puberty.

Coxa valga developed on both sides at age 10 and at age 11 she suffered from increasing tenderness and stiffness of her joints, especially of the fingers. At age 11, she was also diagnosed with primary hypothyroidism.

At age 14, the patient was hospitalized with signs of cerebral ischemia, presenting with right-sided hemiparesis. A cranial CT scan showed signs of infarction of the left medial cerebral artery. In addition, calcifications of the basal ganglia and of the frontal white matter were noticed. MR angiography revealed a complex vascular pathology with complete occlusion of the left distal internal carotid artery and of the medial cerebral artery (Figure 1B). On the right side, there were severe stenoses of the internal carotid artery and of the proximal part of the medial cerebral artery.

Also at age 14, glaucoma of the left eye was diagnosed. Furthermore, a benign paravertebral perineuroma on the left side was detected.

A cerebral MRI scan, performed at age 17, showed marked cerebral atrophy. There were hyperintense lesions, interpreted as calcifications. Presumably resulting from the previous stroke, white matter lesions were evident in the left temporal lobe.

At age 20, her height was 147 cm (3^rd^ percentile), weight was 65 kg (90^th^ percentile) and head circumference 51 cm (3^rd^ percentile). CSF analysis showed no pleocytosis, but increased protein and pterin levels (neopterin 161.1 nmol/l [9-20], biopterin 41.2nmol/l [10-30]) with mild dysfunction of the blood-brain barrier (CSF-to-serum albumin ratio 0.015 [< 0.006]). IFN-α levels were normal in CSF and serum.

## MATERIALS AND METHODS

### Homozygosity mapping

We used standard methods to isolate genomic DNA from peripheral blood samples according to the manufacturer's instructions (Puregene, Gentra Systems, Minneapolis, Minnesota, USA).

From each of the four patients, a DNA sample was processed according to the manufacturer's instructions (Affymetrix GeneChip Human Mapping 50K Assay Manual; Affymetrix, Santa Clara, California, USA). In brief, 250 ng of high quality genomic DNA was digested with HindIII and ligated to HindIII-adaptors. After PCR amplification, random fragmentation and labelling, samples were hybridised to the 50K array (GeneChip Human Mapping 50K Xba 240 array; Affymetrix) in a hybridisation oven (Affymetrix Hybridisation Oven 640; Affymetrix). Washes and staining of the arrays were performed using a fluidics station (Affymetrix Fluidics Station 450; Affymetrix), and images were obtained using a gene-chip scanner (Affymetrix GeneChip Scanner 3000; Affymetrix). Call rates had to exceed 98%. Allele calls were made using the BRLMM (Bayesian Robust Linear Model with Mahalanobis distance classifier) algorithm.

Parametric linkage analysis was performed using the program ALLEGRO (Gudbjartsson et al., 2000) assuming recessive inheritance with complete penetrance and no phenocopy. The disease allele frequency was set to 0.01%. Of all the 58960 SNP markers of the 50K array, we selected 12226 markers for the genome scan based on their minor allele frequencies and their distance to adjacent markers. The filtering was necessary to avoid markers in linkage disequilibrium to each other, which could give rise to false positive signals.

Haplotypes on chromosome 20 were reconstructed by the program ALLEGRO (Gudbjartsson et al., 2000) with microsatellite genotype data of all family members. Fragment analysis for microsatellite genotyping was performed on an ABI 3730 sequencer after PCR amplification with fluorescence labelled primers. Three of the markers had been established at the Cologne Center for Genomics and were registered in the UniSTS and GenBank databases as M20EK01= UniSTS:1233243/GF102162, M20EK02= UniSTS: 1233244/ GF102163 and M20EK03= UniSTS:1233245/GF102164.

All data handling was performed using the user interface ALOHOMORA (Rüschendorf et al., 2005). Pedigree and haplotype drawing was performed with HaploPainter (Thiele et al., 2005).

### DNA enrichment and next generation sequencing

To find the mutation, we used a strategy of DNA enrichment for the region of interest on chromosome 20 followed by complete sequencing using next generation sequencing technology. The sequence capturing was performed with two custom designed NimbleGen 385K Arrays from NimbleGen/Roche, each of them carrying 385,000 oligo-DNA features of 60 bp length. The array design was made to cover a region of 8.2 Mb on chromosome 20 at the positions 30.95 Mb - 39.15 Mb – distributed over two arrays with the sequence data from UCSC human build hg18 and design algorithm 2.0 (http://www.nimblegen.com/products/lit/probe_design_ 2008_06_04.pdf).

In brief, the enrichment process started with fragmentation of 21 μg DNA per array by nebulisation into pieces of 250 bp to approximately 1 kb in size, followed by ligation of 22mer gSel3/gSel4 linkers for a post-enrichment library amplification step. The libraries were then hybridized to the NimleGen sequence capture arrays and subsequently unbound DNA fragments were washed away. After elution and recovery of bound fragments, the enriched pools were checked for quality and were amplified.

For sequencing on the Roche 454 Genome FLX sequencer, DNA libraries were constructed using the Roche shotgun library preparation method for low molecular weight DNA without prior nebulisation (GS FLX General Library Preparation Method Manual, Roche). In a next step, emulsion PCR amplification was performed as described in the Roche Manual (GS FLX Titanium emPCR Method Manual, Roche). The two libraries were then loaded onto a single 2-lane gasket PicoTiterPlate device (70 × 75 mm) and sequenced on a Roche GS FLX sequencer using standard Roche protocols.

Data analysis was performed using Roche's Newbler assembler V.2.0.0 -PostRelease-1 on the basis of human UCSC build hg18. An extended mapping of variants was performed on the ENSEMBL human build V.54_36p with a software developed at the Cologne Center for Genomics to rank variants according to their presumed functional relevance.

### Sanger sequencing

The primer design for the amplification of exonic *SAMHD1* regions was made by GeneExplorer (http://portal.ccg.uni-koeln.de/geneexplorer/). Cleaning of PCR fragments was performed with 3 U Exonuclease I and 1 U Shrimp Phosphatase in a total volume of 10 ul for 20 minutes at 37 °C followed by an inactivation step for 15 minutes at 85 °C. Cycle sequencing was performed with Applied Biosystems BigDye® Terminator v1.1 according to the vendor protocol (http://docs.appliedbiosystems.com/pebiodocs/04337036.pdf). Sequencing runs were performed on an ABI 3730 sequencer.

### Nomenclature

Nucleotide numbering reflects cDNA numbering with +1 corresponding to the A of the ATG translation initiation codon in the reference sequence, according to journal guidelines (http://www.hgvs.org/mutnomen).

### Cell culture

Human skin fibroblasts were isolated from patients and controls after informed consent. The cells were cultured at 37 °C in 5% CO_2_ in MEM with Earl's Salt (PAA Laboratories, Pasching, Austria) supplemented with 10% fetal bovine serum, 2 mM L-glutamine, 200 IU/ml penicillin and 100 μg/ml streptomycin (all from Biochrom, Berlin, Germany). Confluent monolayers of human fibroblasts were stimulated for 4 and 24 hours with 50 μg/ml polyinosinic-polycytidylic acid (Poly(I:C)) or 10 ng/ml tumor necrosis factor alpha (TNF-α) (both from Sigma, Munich, Germany). Control cultures were left untreated.

### Real-time RT-PCR

RNA of control fibroblasts and of patient fibroblasts stimulated with Poly(I:C) was analysed in duplicate. cDNA was synthesized from 1 ug of total RNA using SuperScript™II RNase-H reverse transcriptase (Invitrogen). Primers used for PCR analysis were as follows: *GAPDH* forward, 5′-TGCACCACCAACTGCTTAGC-3′; *GAPDH* reverse, 5′-GGCATGGACTGTGGTCATGAG-3′; *SAMHD1* forward, 5′-AAAACCAGGTTTCACAAC-TTCTGC-3′; *SAMHD1* reverse, 5′-TGCGGCATACAAACTCTTTCTGT-3′; *IFNB1* forward, 5′-TCTGGCACAACAGGTAGTAGGC-3′; *IFNB1* reverse, 5′-GAGAAGCACAACAGGAGAGCAA-3′; *IL8* forward, 5′-CTTGTTCCACTGTGCCTTGGTT-3′; *IL8* reverse, 5′-GCTTCCACATGTCCTCACAACAT-3′. Real-time RT-PCR was performed using the QuantiTect SYBR Green PCR kit (Qiagen, Hilden, Germany) as previously described (Ehrchen et al., 2007). Results are expressed as a number of relative mRNA copies of each gene/10000 mRNA copies of *GAPDH*.

### Western blotting

Fibroblasts were trypsinized, washed in PBS and lysed in High Salt Lysis Buffer (20 mM Hepes pH 7.9, 420 mM NaCl, 1.5 mM MgCl_2_ and 0.2 mM EDTA) supplemented with EDTA-free complete protease inhibitors (Roche, Mannheim, Germany). The protein concentration of cell lysates was determined by the Bradford method. Equivalent amounts of protein (20 μg/lane) were subjected to electrophoresis on a 10% sodium dodecyl-sulfate acrylamide gel and subsequently electroblotted to a nitrocellulose membrane (Pierce, Rockford, IL, USA). The membranes were probed with rabbit antibody against SAMHD1 (Protein Tech Group, Chicago, IL, USA) followed by horseradish peroxidase-coupled secondary antibody using enhanced chemiluminescence. Membranes were directly reprobed with mouse monoclonal anti-β-actin antibody to verify equal loading and transfer of proteins (Sigma, Munich, Germany).

### Immunofluorescence microscopy

Fibroblasts were cultured at a concentration 40 × 10^3^ cells/ml on fibronectin (Becton Dickinson)-coated LabTec chamber slides (Nunc, Wiesbaden, Germany) to determine the cellular localization of SAMHD1 protein. Cells were stimulated with Poly(I:C) for 24 h or left untreated. Subsequently, cells were fixed with 10% paraformaldehyde in PBS for 10 minutes, permeabilized with pre-chilled acetone (-20 °C) for 5 minutes and washed in PBS. After blocking of unspecific binding sites with 1% BSA in PBS at room temperature for 1 h, staining with anti-SAMHD1 antibody (ProteinTech Group, Chicago, IL, USA) was performed. As secondary antibody FITC-conjugated goat anti-rabbit IgG (Dianova, Hamburg, Germany) was used. Cell nuclei were visualized by staining with 4,6-diamidino-2-phenylindole (DAPI) (Sigma, Munich, Germany). Coverslips were mounted on slides using Fluorescent Mounting Medium (Sigma, Munich, Germany). Immunofluorescence analysis was conducted using Carl Zeiss Axio Observer.Z1 microscope with AxioCam MRm camera and EC Plan-Neofluor Oil immersion (x40) lens using Axio Vision Software.

## RESULTS

### Locus Mapping

**Figure 2:**
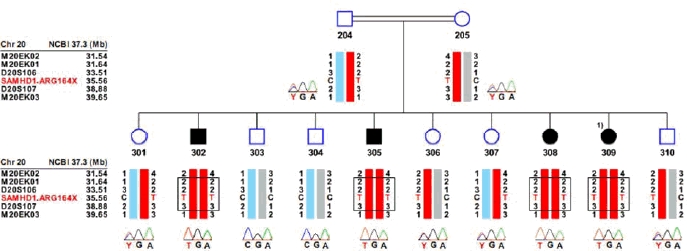
The *SAMHD1* Arg164X mutation cosegregates with the red haplotypes of the family. A haplotype analysis with microsatellite genotype data of the main region of interest further confirmed the locus on chromosome 20. Only the four affected family members were homozygous for the markers in the interval as represented by the surrounding box. The marked individual 1 was chosen for sequencing of the whole region by next generation sequencing technology after DNA enrichment. The stop mutation CGA > TGA found in Arg164 of the *SAMHD1* gene (GenBank NG_017059.1) was validated by Sanger sequencing. The figure also integrates the red colored mutation as part of the red colored disease haplotypes. Mapping coordinates are given in physical distances according to human NCBI build 37.3.

To map the disease locus, a genome-wide linkage analysis was performed using the four affected family members. We found only one prominent linkage signal in the genome, namely on chromosome 20, showing a multipoint LOD score of 3.0 (data reviewed but not shown). The locus was confirmed by genotyping of microsatellite markers in all family members. Segregation of haplotypes of the critical interval on chromosome 20 is shown in Figure 2. Only the four patients were homozygous for the disease carrying haplotype in the linkage interval. The flanking markers M20EK02 and M20EK03 defined an interval of about 8.1 mega bases, where the mutation had to be looked for.

### Sequence capturing and next generation sequencing

After masking of regions with low complexity, a total of 6.18 mega bases (Mb) of sequences could finally be used for the NimbleGen 385K array design. This represents a coverage of 75.3%. For the first library, 470,659 sequence reads comprising 135 Mb of sequences were recoverable. About 76% of the sequences could be mapped to the target region resulting in a total target coverage (>1x) of 85%. The second library resulted in 650,943 reads and 199 Mb of sequences (65% on target with 86% target coverage). In total, 231 Mb of sequences covering the target region were available for mutation screening. With respect to coverage distribution, 29% and 24% of the mapped region were underrepresented with a factor of < 6 for the two enrichments.

Overall, 16,454 variants could be identified, most of them representing false positive signals due to low quality of sequence data. As a homozygous mutation was expected, a primary filter for variants with a frequency of at least 50% reduced the list to 6721. 755 variants remained after elimination of known SNP variants. Only 19 of these variants mapped to protein coding transcripts and led to an amino acid change as well. 5 variants were found to overlap with potential splice sites. By increasing the variant frequency limit to 90%, a total of 5 different amino acid changes in 4 different genes remained (reviewed but not shown) – one of them being the nonsense mutation c.490C>T (p.Arg164X) inexon4 of the *SAMHD1* gene (ENSP00000262878, GenBankNG_0 17059.1), which was seen in both enriched libraries in 12 of 13 reads.

### Mutation screening

To validate the *SAMHD1* c.490C>T (p.Arg164X) mutation, all family members were sequenced with the Sanger method. The mutation could be confirmed in all family members segregating the disease haplotype with the patients showing a homozygous mutation, while the parents and some of the unaffected siblings presented as heterozygous carriers (Figure 2). The c.490C>T (p.Arg164X) mutation could not be found in 96 Sanger sequenced Caucasian control samples.

### SAMHD1 expression

**Figure 3:**
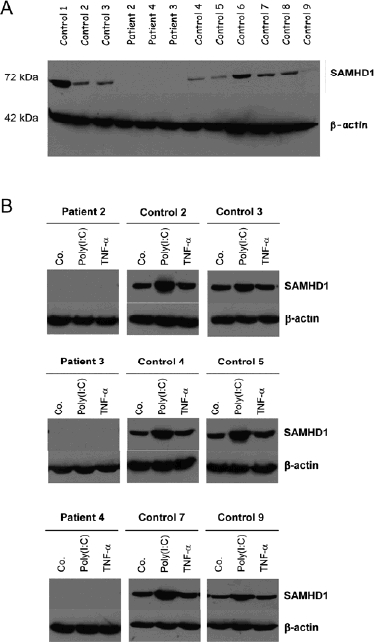
Expression of SAMHD1 protein in human fibroblasts. (A) Western blot with antibody recognizing SAMHD1 protein. Blot was reprobed with β-actin antibody to control for equal loading. (B) Fibroblasts treated with Poly(I:C) and TNF-α for 24 h, lysed and immunoblotted using SAMHD1 antibody. Anti-β-actin antibody was used as a loading control. Co., Control. Poly(I:C), polyinosinic-polycytidylic acid. TNF-α, tumor necrosis factor alpha.

*SAMHD1* mRNA was present in all control fibroblasts as well as in fibroblasts isolated from patients with the p.Arg164X substitution, as assessed by real-time RT-PCR and dissociation curve analysis (data not shown). Analysis of protein expression in Western blot showed that SAMHD1 protein was expressed in all control fibroblasts, but was not detectable at protein level in lysates of fibroblasts isolated from affected individuals (Figure 3A).

### Changes in SAMHD1 expression in response to Poly(I:C) and TNF-α

Western blots were performed to evaluate changes in expression of SAMHD1 protein in response to pro-inflammatory stimuli. Fibroblasts were stimulated with Poly (I: C) and TNF-α for 24 h. We found significantly increased expression of SAMHD1 in lysates of control fibroblasts treated with Poly(I:C) i.e., a 1.5 to 2.2-fold induction as revealed by densitometric quantification of SAMHD1 expression normalized to β-actin (data not shown), while the amount of SAMHD1 protein was not changed or only very slightly up-regulated after treatment with TNF-α. SAMHD1 protein was not detectable in lysates of patient fibroblasts (Fig. 3B).

Immunocytochemistry confirmed these observations. SAMHD1 protein was detectable in unstimulated control fibroblasts and its expression was increased in response to Poly(I:C) stimulation. Both in unstimulated and Poly(I:C)-stimulated cells SAMHD1 protein was mainly localized to the nucleus. In contrast, SAMHD1 protein was not detected in patient fibroblasts (data not shown).

### The role of SAMHD1 protein in immune response against viral infection

**Figure 4:**
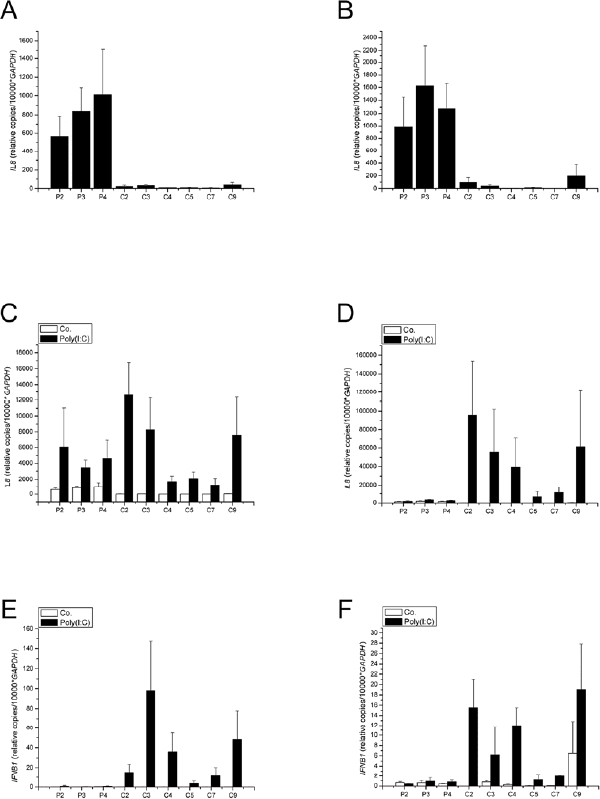
Time-course studies of the fibroblasts' response to Poly(I:C). Basal expression of *IL8* after 4 (Panel A) and 24 hours (Panel B). Expression of *IL8* after stimulation with Poly(I:C) after 4 (Panel C) and 24 hours (Panel D). Expression of *IFNB1* after stimulation with Poly(I:C) after 4 (Panel E) and 24 hours (Panel F). The plots show mean ± SEM (n=3). Differences between patients and controls regarding *IL8* and *IFNB1* expression are statistically significant (p<0.024, Mann-Whitney test). Control fibroblasts are numbered C2 to C9, patient fibroblasts P2 to P4. Co., Control. *IL8*, interleukin-8. *IFNB1*, interferon-beta. Poly(I:C), polyinosinic-polycytidylic acid.

We treated control fibroblasts and fibroblasts isolated from affected individuals with the TLR3-agonist and immunostimulant polyinosinic:polycytidylic acid (Poly(I:C)) to investigate how lack of SAMHD1 protein modifies immune response. Fibroblast response to Poly(I:C) was assessed by measuring the expression of pro-inflammatory factors using quantitative real-time RT-PCR. We did not detect any differences in basal and Poly(I:C)-induced expression of interleukin-6 *(IL6)*, chemokine (C-X-C motif) ligand 9 *(CXCL9)* and intercellular adhesion molecule 1 *(ICAM1)* (data not shown). However, we found highly up-regulated basal expression of interleukin-8 *(IL8)* in patient fibroblasts as compared to controls (Figure 4A and 4B). After 4 h of stimulation with Poly(I:C), similar amounts of *IL8* transcript were induced in control fibroblasts and patient fibroblasts (Fig. 4C). After 24 h of Poly(I:C)-stimulation, *IL8* was still up-regulated in control fibroblasts, but not in patient fibroblasts (Fig. 4D). High amounts of interferon-beta *(IFNB1)* transcript were found in control fibroblasts after 4 h of stimulation with Poly(I:C) (Fig. 4E) and up-regulation was still present after 24 h (Fig. 4F). *IFNB1* was not induced in response to Poly(I:C) in patient fibroblasts (Fig. 4E and 4F).

## DISCUSSION

We report on four siblings born to consanguineous parents of Turkish origin affected by a neurodegenerative, multisystemic disease. The patients presented with psychomotor retardation, spasticity, short stature, skin lesions and, most notably, with a triad of stenoses of intracranial vessels, stroke, and glaucoma. Three of these patients had been treated for a multisystemic inflammatory autoimmune process for many years. However, we had been unable to link their symptoms to a known syndrome. Suspecting a hitherto undescribed entity, we performed homozygosity mapping to identify its genetic cause. The genome-wide linkage analysis showed a multipoint LOD score of 3.0 on chromosome 20. A homozygous region spanning 8.1 Mb and harboring more than 170 candidate genes was detected in the four affected siblings. The complete candidate region was then sequenced after enrichment using next generation sequencing technology. The nonsense mutation c.490C>T (p.Arg164X) in exon 4 of the *SAMHD1* gene was rapidly identified. The four affected patients were found to be homozygous carriers of this mutation, as expected. *SAMHD1* mRNA was expressed in patient fibroblasts, but SAMHD1 protein was absent in patient fibroblasts. Furthermore, patient fibroblasts showed a modulation of the cytokine expression with elevation of basal expression of interleukin-8 *(IL8)*. Interleukin-8 is a cytokine produced by phagocytes and mesenchymal cells exposed to inflammatory stimuli (e.g., interleukin-1 or tumor necrosis factor) and activates neutrophils by inducing chemotaxis, exocytosis and the respiratory burst (Bagiolini and Clark-Lewis 1992). Additionally, stimulated expression of interferon-beta *(IFNB1)* was reduced. Interferon-beta is a dominant factor in shaping downstream events in the innate and adaptive immune responses subsequent to viral infections (Theofilopoulos et al., 2005).

While we carried out our study, *SAMHD1* was described as a disease-causing gene in AGS (Rice et al., 2009). We therefore retrospectively searched for markers supporting the diagnosis of AGS in our patients' medical files and completed diagnostic studies in the two surviving patients. Intracerebral calcifications of the basal ganglia were present on CT in all four patients. Reevaluation of the two surviving patients revealed elevated pterine levels in CSF, while cell counts and IFN-α levels in CSF and IFN-α levels in plasma were normal. Diagnosis of AGS in our four patients was thus confirmed.

AGS had been considered as differential diagnosis in this family because of the presence of intracerebral calcifications. The diagnosis was discarded, however, because the milder phenotype and the triad of stenoses of intracranial vessels, stroke, and glaucoma were considered to be clinically incompatible with the disease. In light of our findings, we conclude that the features present in our patients have to be regarded as an extension of the phenotype of AGS. Interestingly, the p.Arg164X mutation found in our patients was reported most recently in combination with the mutation c.433C>T (p.R145X) in two patients with AGS, who suffered from chronic arthropathy and contractures (Dale et al., 2010). These symptoms were also present to a varying degree in our patients. However, our patients additionally presented with precocious puberty (all patients), calcinosis cutis (patient no. 4) and intracranial “large vessel disease” (patient no. 1, no. 2, and no. 4) not previously reported in AGS patients.

The *SAMHD1* gene comprises of 16 exons and encodes a 72.2 kDa protein consisting of 626 amino acid residues. The SAMHD1 protein contains a SAM (sterile alpha motif) domain and a HD (histidine / aspartic acid) domain. SAMHD1 was initially designated as dendritic cell-derived IFN-γ induced protein (DCIP) (Li et al., 2000) since it was identified in monocyte-derived dendritic cells as a human homologue of mouse IFN-γ induced protein. Overall, mutations in *SAMHD1* seem to result in a milder phenotype of AGS compared to mutations in *TREX1* and *RNASEH2* and *RNASEH2C* (Rice et al., 2009). This is supported by our study, as the patients presented here survived into adulthood and one affected patient showed only mild cognitive impairment.

Pathophysiologic mechanisms associated with mutations in *SAMHD1* and their relation to mutations in *TREX1* or *RNASEH2* are not fully understood (Crow and Rehwinkel, 2009). TREX1 and RNASEH2 are involved in DNA and RNA degradation. Defective TREX1 and RNASEH2 protein complex activities lead to accumulation of DNA and RNA by-products, mimicking viral infection and causing antiviral immune response (Alarcon-Riquelme, 2006; Bhoj et al. 2008). SAMHD1 is upregulated in viral infection (Préhaud et al., 2005; Zhao et al., 2008) and is induced by TNF-α (Liao et al., 2008). Its exact function, however, needs to be further elucidated.

In our patients, SAMHD1 was not expressed when mimicking viral infection in vitro using the Toll-like receptor-3 (TLR3)-agonist Poly(I:C). When stimulated with Poly(I:C), there was also no expression of interferon-beta *(IFNB1)*, a cytokine present in viral infection. Low expression of *IFNB1* in fibroblasts of AGS-patients was an unexpected finding. However, this pattern was found in cells obtained from independent patients and was highly reproducible in several independent sets of experiments. Although our data point to a dysregulation in the stress response to inflammatory stimuli, several reasons may explain the lack of *IFNB1* up-regulation in fibroblasts in vitro. It is well known that the interferon (IFN) pathway is induced by TLR-dependent as well as by TLR-independent pathways. In our experimental setting only TLR-dependent signalling cascades are activated. Initial TLR-independent activation pathways, however, have been supposed to be highly relevant for autoimmune processes (Baccala et al., 2007). In addition, an emerging paradigm in innate immunity signalling is that the biological context of the cell, e.g. the intracellular compartimentalization, can influence the outcome of a ligand-receptor interaction, which may differ between the situations in vivo and our experimental setting in vitro (Barton and Kagan, 2009). Last but not least, several groups have identified a pattern of enhanced expression of genes, which are regulated by type I interferons in patients with systemic lupus erythematosus. These findings have shown that mainly myeloid and plasmacytoid dendritic cells, but not fibroblasts, are the relevant sources of interferons in this autoimmune disease (Croker and Kimberly, 2005). Thus, high interferon-beta levels found in AGS patients may be due to secondary immune mechanisms during the later course of disease. Therefore, our finding of reduced *IFNB1* expression in patient fibroblasts might not reflect the complex pathology of AGS in vivo.

Moreover, basal expression of interleukin-8 *(IL8)*, a potent chemoattractant for neutrophils and a central mediator of vascular inflammation, was increased. This seems to be very specific, since other pro-inflammatory molecules like IL-6, ICAM-1 or CXCL9 were not differentially regulated. Interleukin-8 plays a crucial role in initiating atherosclerosis by recruiting monocytes and macrophages to the vessel wall (Ito and Ikeda, 2003) and has been shown, together with IL-6 and RANTES, to be elevated and to correlate with disease activity in patients with Takayasu's arteritis (Arnaud et al., 2006). Although intracranial stenoses of large cerebral arteries have not been reported previously in AGS, inflammatory microangiopathy is present in a subset of patients with AGS (Barth, 2002). Neurovascular pathology has also been observed in patients with Retinal Vasculopathy and Cerebral Leukodystrophy (RVCL) and with Hereditary Endotheliopathy, Retinopathy and Nephropathy (HERNS) caused by *TREX1* mutations (Kavanagh et al., 2008). We speculate that increased IL-8 levels contribute to the vascular pathology in AGS. However, at this point, it is not clear, whether overproduction of IL-8 in patient fibroblasts is unique to patients with *SAMHD1* mutations or whether this is a shared feature in all AGS patients.

In summary, the Arg164X mutation in *SAMHD1* was associated with stenoses of intracranial vessels, stroke, and glaucoma, a finding extending the phenotype of AGS. Moreover, our data show a dysregulation of the primary inflammatory stress response in our patients' fibroblasts. It may be speculated that this mechanism may be involved in the vascular pathology of the patients presented here.
